# Artificial Intelligence and Machine Learning in Endocrinology and Metabolism: The Dawn of a New Era

**DOI:** 10.3389/fendo.2019.00185

**Published:** 2019-03-28

**Authors:** Sriram Gubbi, Pavel Hamet, Johanne Tremblay, Christian A. Koch, Fady Hannah-Shmouni

**Affiliations:** ^1^Diabetes, Endocrinology, and Obesity Branch, National Institute of Diabetes, Digestive and Kidney Diseases, National Institutes of Health, Bethesda, MD, United States; ^2^Centre de Recherche, Centre Hospitalier de l'Université de Montréal, Montréal, QC, Canada; ^3^Département de Médecine, Université de Montréal, Montréal, QC, Canada; ^4^Medicover GmbH, Berlin, Germany; ^5^Department of Medicine, Carl von Ossietzky University, Oldenburg, Germany; ^6^University of Tennessee Health Science Center, Memphis, TN, United States; ^7^Section on Endocrinology and Genetics, National Institute of Child Health and Human Development, National Institutes of Health, Bethesda, MD, United States

**Keywords:** artificial intelligence, machine learning, diabetes, thyroid, endocrinology, metabolism

The rapid growth of technology in the past couple of decades has paved the way for development of novel techniques that can solve scientific questions at a rate that is far beyond the capability of humans. One such example is the field of Artificial Intelligence (AI) and Machine Learning (ML). AI is a discipline that deals with the study and design of intelligent agents, that is, devices that intricately perceive their environment and take actions that maximize the chances of achieving their goals (1). AI, in a way, mimics the structure and operating methodologies of a human brain (2). AI has two forms of application: physical and virtual (3). The physical component is mainly represented by robots. Derived from a Czech word *robota*, meaning “forced labor,” the physical robotic forms were conceptualized by inventors such as Leonardo Da Vinci (3). This component has been widely used in the field of endocrinology, such as robot-assisted surgery of adrenal or prostate cancer. Examples of virtual applications of AI are electronic medical records (EMR), where specific algorithms are used to identify subjects, and harness health related data (3).

ML is a field of AI that deals with the development of models and intricate networks that enable computer systems to improve their performance on a specific task progressively (4). ML algorithms can be: (i) unsupervised (spontaneous pattern detection), (ii) supervised (building algorithms based on prior examples), or (iii) reinforcement learning (utilization of reward/punishment techniques to obtain the desired result) (3). A common use of ML in daily life includes flagging spam in an e-mail, autonomous driving and selecting the best route for daily commute. In the field of medicine, AI/ML technology can have substantial impact at three levels: physicians, by improving the diagnostic accuracy and assisting with therapeutic and surgical interventions; health systems, by enabling improved workflow and reduction in errors; patients, through tailoring of diagnostic, and treatment modalities based on the unique phenotypic and genetic features of individual patients (5). In this review, we focus on the virtual components of AI and ML and provide some examples for the utility of AI/ML in endocrinology and metabolism.

From early ML tools like logistic regression which found their utility in medicine several decades ago, AI/ML methods have become far more multifaceted and have revolutionized the field of medicine through their ability to compute and analyze vast and complex array of datasets which would not be feasible solely with trained human skillsets (2). Several AI/ML methods have proven their utility in the diagnosis and management of various endocrinopathies. Gradient forest analysis, a ML technique, was applied in a study to identify factors contributing to variation in all-cause mortality among subjects in the Action to Control Cardiovascular Risk in Diabetes (ACCORD) trial (6). This technique detected four risk groups based on hemoglobin glycosylation index (HGI), BMI, and age. The lowest risk group (with HGI < 0.44, BMI < 30 kg/m^2^, and age <61 years) experienced reduced absolute mortality risk of 2.3%, while the highest risk group (HGI > 0.44) experienced a 3.7% increase in absolute mortality risk attributable to intensive glycemic therapy. These mortality variations in the intensive treatment group were previously not detected by older, univariate subgroup analyses (6). Another study developed a prototype support vector regression model that outperformed diabetologists in predicting blood glucose levels at 30 and 60 min from a given time in patients with type 1 diabetes, and predicted about one quarter of hypoglycemic events 30 min ahead of the actual event (7).

AI/ML-based algorithms have been extensively utilized and validated for diagnosis and classification of diabetic retinopathy (8–11). Deep learning systems and even purely database-driven AI algorithms have demonstrated the ability to diagnose diabetic retinopathy and related retinal diseases in large, multiethnic cohorts with high degree of sensitivity and specificity (8, 10). ML algorithms have also demonstrated the ability to incorporate associated risk factors such as duration of diabetes and insulin use into risk-stratification of diabetic retinopathy, which could potentially facilitate the development of better clinical decision support systems (11). A proprietary system IDx (Iowa City, IA) that uses ML technology to analyze retinal images in diabetic retinopathy had a sensitivity of 87% and specificity of 91% for autonomous detection of disease and received FDA approval in 2018 (12). This example represents the first prospective assessment of AI/ML in the clinic (5).

AI/ML technologies has also been used in the analysis of large datasets generated from genomic technology. Deep-coverage whole-genome sequencing performed in 8,392 individuals of European and African descent to identify single-nucleotide variants and copy-number variations in Lipoprotein (a) revealed that *LPA* risk genotypes conferred greater relative risk for incident cardiovascular disease than direct measurements of Lipoprotein (a) levels. These risk genotypes were also associated with increased sub-clinical atherosclerotic disease in individuals of African ancestry (13). ML techniques were utilized in developing a novel mRNA based molecular test to detect *BRAF* V600E mutations in thyroid fine needle aspirate samples, which demonstrated sensitivity equal to that of established DNA-based assay and had lower non-diagnostic rates (14). By utilizing functional enrichment analysis followed by module analysis performed on protein-protein interaction network, the differential gene expression in anaplastic thyroid carcinoma was assessed (15). There were 247 up-regulated genes which were predominantly involved in cell cycle and 275 down-regulated genes that were mostly involved in thyroid hormone synthesis, insulin resistance, and cancer pathways, thus expanding on the current knowledge of genetics of thyroid carcinomas (15).

AI/ML methods can accurately interpret medical images and provide a computer-aided diagnosis. ML algorithms like convolutional neural network and support vector machine (SVM) demonstrated higher sensitivity, specificity, and positive and negative predictive values with early detection of facial changes in acromegaly when compared with doctors' evaluation, thus allowing for earlier clinical diagnosis and evaluation (16). A simple diagrammatic representation of utilization of facial landmark recognition for diagnosis of acromegaly is provided in Figure 1. ML has enabled individuals to make optimal decisions in real time by enabling organizational performance (3). For example, utilization of ML techniques such as principal component analysis and SVM, and matrix-assisted laser desorption/ionization mass spectrometry (MS) imaging demonstrated the potential to differentiate hormone-secreting from non-secreting pituitary adenomas, and demarcate tumor from normal gland at a molecular level under 30 minutes, therefore potentially enabling real-time, intra-operative tumor delineation, and improve patient outcomes (17).

**Figure 1:**
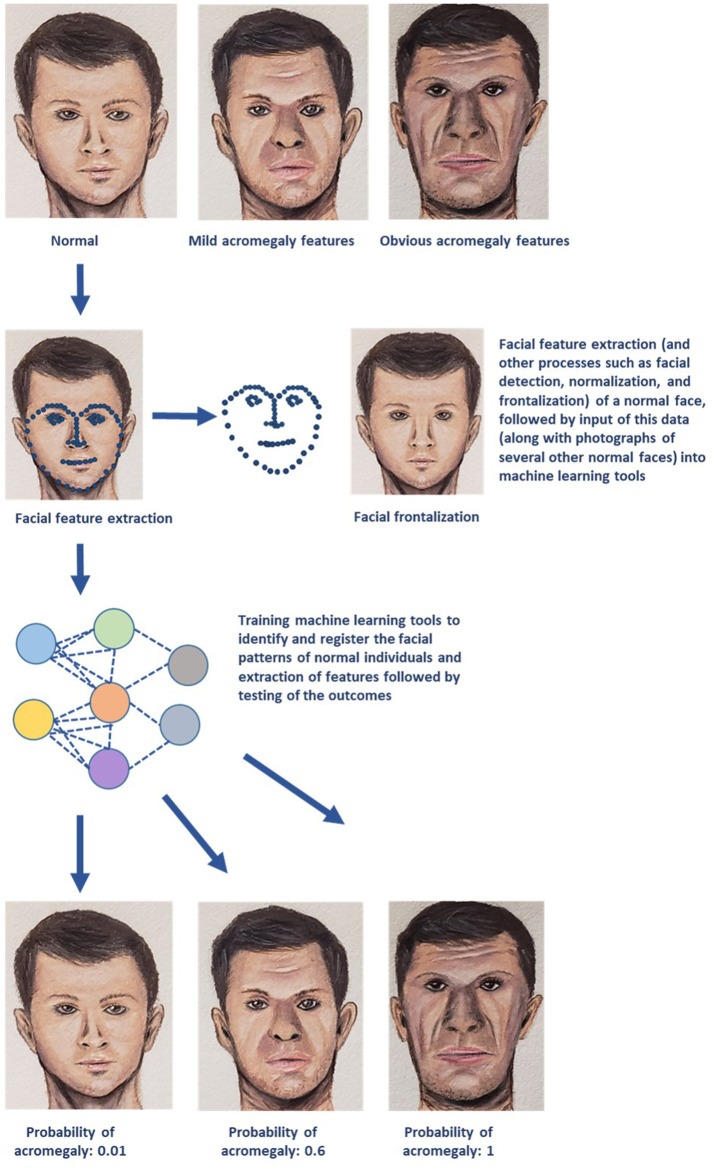
A simplified schematic representation of one of the several machine learning (ML) technologies that can be utilized for diagnosis and management of endocrine disorders. The example demonstrated in the above diagram deals with early recognition of acromegaly based on facial features. Photographs of several individuals with normal facial features are utilized to obtain data on facial patterns through processes such as facial feature extraction (represented by blue dotted lines), facial detection, normalization, and frontalization (also displayed in the figure). This data is then fed into ML algorithms for recognizing normal facial features. These ML tools then perform complex analyses and generate output data that is used to determine whether the face presented in the test photograph is consistent with features of acromegaly (Image courtesy: Sriram Gubbi, NIDDK, NIH).

Variants of SVM, neural networks, and other ML techniques, with immunohistochemical methods were utilized in categorizing Cushing syndrome with adrenocortical lesions (18). Based on the gene expression profiling, the highest expressions of Ki-67 and PCNA were found in adrenocortical carcinoma while highest FHIT expression was found in adrenocortical hyperplasia, and adrenal adenomas had intermediate expression of all three antigens. These techniques diagnosed the adrenocortical disease type with 92.6% accuracy. In another study, urinary steroid profiling using gas chromatography/MS and subsequent ML analysis generated a pattern of immature, early stage steroid metabolites in adrenocortical cancer (19). This enabled differentiation of cancer from an adenoma with a sensitivity and specificity of 90%, a diagnostic value that was higher than CT, MRI, or PET scans.

Another emerging field based on AI technology that could potentially have a wider scope in the future is “pre-emptive medicine” (20). Pre-emptive medicine is a novel concept proposed in Japan, which aims at delaying the onset, or even preventing the occurrence of chronic diseases, such as diabetes, hypertension, cancer, or dementia by using a combination of AI techniques, genomic analysis and environmental interaction data (20). The above examples reinforce the promising role of AI/ML in diagnosis and management of endocrine disorders which, in several instances, can outperform skilled physicians, minimize resource use and allocation, and yield tangible benefits by supporting physicians and accelerating clinical decision-making (7, 16). Despite the substantial evidence for the ability of AI/ML to deliver cost-effective healthcare and improve patient outcomes, medicine has trailed behind other scientific fields in implementing these techniques into practice (21). Potential hurdles include the longitudinal nature of variations in human disease, inadequacies in the quality and reliability, heterogeneity of healthcare data, personal data confidentiality, need for informed consent from patients, requirement of supportive policies and efficient business models, unpredictable reimbursement, and increasing necessity for data sharing (21, 22). The so-called “digital biomarkers” that are obtained through big data analyses performed using AI/ML techniques are not readily interpretable clinically, in the sense, even if a certain newer AI/ML algorithm has been shown to be superior to older techniques in certain population cohorts; its implementation in clinical practice across more diverse populations might not necessarily result in better diagnosis or outcome; and could potentially even lead to over-diagnosis and over-treatment in certain patient cohorts (23). Ethical issues, including misuse of AI/ML to manipulate quality metrics to make unscrupulous profit, potential in-built discriminatory biases toward under-represented populations, and physician over-dependence on AI/ML pose some of the foreseeable challenges in this field (24). Although AI/ML can theoretically “replace” physicians with regards to performing certain diagnostic, therapeutic, or surgical tasks, these technologies, almost certainly, will never be able to provide the emotional, social, and ethical support that a physician can offer the patient, and will never replace the unique bond of a doctor-patient relationship (25, 26).

Then the question arises: What aspects of patient care can physicians focus on in the era of AI/ML technology? With the ever-growing complexity and quantity of knowledge in the medical field, it is almost impossible for physicians to mentally organize and retain all of this data (27). Therefore, the medical fraternity should focus on strengthening the following aspects in order to re-define the role of physicians in the era of AI/ML: (1) Medical school curricula must shift their focus from information acquisition to knowledge management and communication skills, (2) Physicians must be trained to manage and collaborate with AI/ML applications, (3) Emphasis must be placed on training physicians to interpret AI/ML output data and to effectively utilize these results in clinical decision making, and (4) Reinforcing cultivation of empathy and compassion among physicians (27). Eventually, physicians and AI technology need to develop a mutually supportive relationship than a competitive one. While physicians can provide appropriate feedback for AI/ML techniques and tools to improve, these tools in turn can facilitate physicians in solving uncertain clinical scenarios (28). This can result in mutual identification of key clinical or algorithmic biases, which can be then tackled by the combined efforts of physicians and AI/ML through better data collection and through model improvements (28).

In conclusion, utilization of AI/ML will enable diagnosing endocrine disorders with higher accuracy, potentially avoid unnecessary investigations, and reduce healthcare expenditures and facilitate better digital storage of vast patient data, be it individual profiles or aggregated data for epidemiological research and planning, and these benefits may one day transform clinical endocrine practice. Today, AI technologies like artificial pancreas for management of diabetes have already become a reality (29). Major efforts are required from academia and the information technology industry to push for further development of AI/ML technology in endocrinology. Endocrinologists are well suited to play a vital role in the advancement of AI/ML. However, this has not been the focus of training programs for endocrine subspecialties, which do not provide the necessary education for trainees to feel confident in the use of these technologies for diagnosis or research. There is certainly a need to spread awareness, acquire funding, introduce these concepts into training programs, and encourage further research in this new, exciting branch of endocrinology and metabolism—a deep future awaits!

## Author Contributions

SG prepared the manuscript and the figure. FH-S, PH, JT, and CK wrote parts of the manuscript and critically reviewed the manuscript.

### Conflict of Interest Statement

The authors declare that the research was conducted in the absence of any commercial or financial relationships that could be construed as a potential conflict of interest.
